# (2*E*)-1-(2,5-Dimeth­oxy­phen­yl)-3-(3-nitro­phen­yl)prop-2-en-1-one

**DOI:** 10.1107/S1600536811043224

**Published:** 2011-10-29

**Authors:** Hoong-Kun Fun, Tze Shyang Chia, B. Narayana, Prakash S. Nayak, B. K. Sarojini

**Affiliations:** a X-ray Crystallography Unit, School of Physics, Universiti Sains Malaysia, 11800 USM, Penang, Malaysia; b Department of Studies in Chemistry, Mangalore University, Mangalagangotri, Mangalore 574 199, India; c Department of Chemistry, P. A. College of Engineering, Mangalore 574 153, India

## Abstract

In the title compound, C_17_H_15_NO_5_, an intra­molecular C—H⋯O hydrogen bond generates an *S*(6) ring motif. The benzene rings form a dihedral angle of 6.45 (7)° with each other. In the crystal, inversion dimers linked by pairs of C—H⋯O hydrogen bonds generate *R*_2_^2^(8) loops. Adjacent dimers are further connected by C—H⋯O hydrogen bonds into an infinite chain along the [011] direction.

## Related literature

For biological activities of chalcones, see: Dimmock *et al.* (1999 ▶). For the structures of chalcone derivatives, see: Samshuddin *et al.* (2010 ▶); Fun *et al.* (2010*a* ▶,*b* ▶); Jasinski *et al.* (2010 ▶); Baktır *et al.* (2011*a* ▶,*b* ▶). For related crystal structures, see: Jasinski *et al.* (2008 ▶); Sarojini *et al.* (2007 ▶); Ma (2007 ▶). For hydrogen-bond motifs, see: Bernstein *et al.* (1995 ▶). For standard bond lengths, see: Allen *et al.* (1987 ▶). 
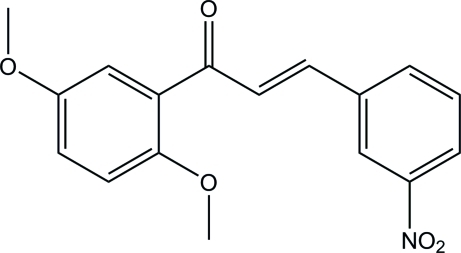


## Experimental

### 

#### Crystal data

C_17_H_15_NO_5_
*M* *_r_* = 313.30
Triclinic, 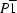

*a* = 7.5015 (5) Å
*b* = 7.9962 (5) Å
*c* = 13.2468 (8) Å
α = 86.507 (1)°
β = 80.342 (1)°
γ = 76.332 (1)°
*V* = 760.96 (8) Å^3^
*Z* = 2
Mo *K*α radiation
μ = 0.10 mm^−1^
*T* = 296 K
0.41 × 0.38 × 0.13 mm

#### Data collection

Bruker APEX DUO CCD area-detector diffractometer
Absorption correction: multi-scan (*SADABS*; Bruker, 2009 ▶) *T*_min_ = 0.960, *T*_max_ = 0.987
16631 measured reflections
4381 independent reflections
3195 reflections with *I* > 2σ(*I*)
*R*_int_ = 0.021

#### Refinement

*R*[*F* ^2^ > 2σ(*F* ^2^)] = 0.048
*wR*(*F* ^2^) = 0.179
*S* = 1.02
4381 reflections
210 parameters
H-atom parameters constrained
Δρ_max_ = 0.25 e Å^−3^
Δρ_min_ = −0.22 e Å^−3^

### 

Data collection: *APEX2* (Bruker, 2009 ▶); cell refinement: *SAINT* (Bruker, 2009 ▶); data reduction: *SAINT*; program(s) used to solve structure: *SHELXTL* (Sheldrick, 2008 ▶); program(s) used to refine structure: *SHELXTL*; molecular graphics: *SHELXTL*; software used to prepare material for publication: *SHELXTL* and *PLATON* (Spek, 2009 ▶).

## Supplementary Material

Crystal structure: contains datablock(s) global, I. DOI: 10.1107/S1600536811043224/is2794sup1.cif

Structure factors: contains datablock(s) I. DOI: 10.1107/S1600536811043224/is2794Isup2.hkl

Supplementary material file. DOI: 10.1107/S1600536811043224/is2794Isup3.cml

Additional supplementary materials:  crystallographic information; 3D view; checkCIF report

## Figures and Tables

**Table 1:** Hydrogen-bond geometry (Å, °)

*D*—H⋯*A*	*D*—H	H⋯*A*	*D*⋯*A*	*D*—H⋯*A*
C3—H3*A*⋯O2^i^	0.93	2.55	3.4773 (18)	172
C8—H8*A*⋯O1	0.93	2.12	2.7727 (16)	126
C17—H17*A*⋯O5^ii^	0.96	2.50	3.309 (2)	142

Symmetry codes: (i) 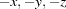; (ii) 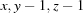.

**Fig. 1.**
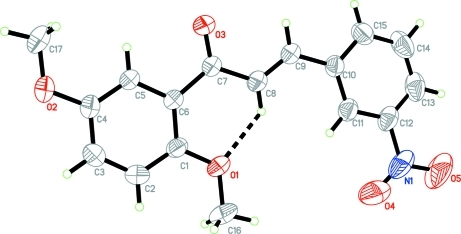
The molecular structure of the title compound with atom labels with 50% probability displacement ellipsoids. The intramolecular hydrogen bond is shown by a dashed line.

**Fig. 2.**
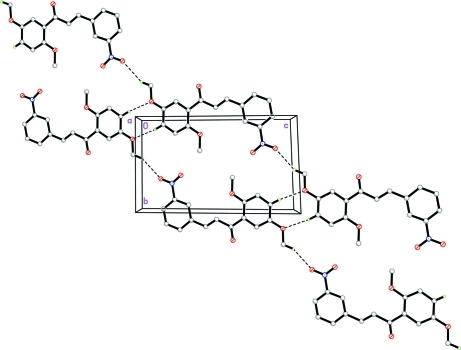
A packing diagram of the title compound viewed along the a axis. The dashed lines represent the hydrogen bonds.

## supplementary crystallographic information

### Comment

Chalcones can be easily obtained from the Claisen–Schmidt reaction of aromatic aldehydes and aromatic ketones. Chalcones have been reported to possess many useful properties including anti-inflammatory, antimicrobial, antifungal, antioxidant, cytotoxic, antitumour and anticancer activities (Dimmock *et al.*, 1999). The basic skeleton of chalcones which possess the α,β-unsaturated carbonyl group is a useful synthone for the synthesis of various biodynamic cyclic derivatives such as pyrazoline, benzodiazepine and cyclohexenone derivatives (Samshuddin *et al.*, 2010; Fun *et al.*, 2010*a*,*b*; Jasinski *et al.*, 2010; Baktır *et al.*, 2011*a*,*b*). The crystal structures of some related chalcones which contain the nitro and methoxy groups *viz*: (2*E*)-3-(4-methylphenyl)-1-(3-nitrophenyl)prop-2-en-1-one (Jasinski *et al.*, 2008), (2*E*)-3-(2-chlorophenyl)-1-(3-nitrophenyl)prop-2-en-1-one (Sarojini *et al.*, 2007) and (*E*)-3-(4-methoxyphenyl)-1-(3-nitrophenyl)prop-2-en-1-one (Ma, 2007) have been reported. In view of the importance of chalcones, the crystal structure of the title compound is reported here.

The molecular structure of the title compound is shown in Fig. 1. The benzene rings (C1–C6 and C10–C15) make a dihedral angle of 6.45 (7)° with each other. Bond lengths (Allen *et al.*, 1987) and angles are within normal ranges and are comparable to related structures (Jasinski *et al.*, 2008; Sarojini *et al.*, 2007; Ma, 2007). The molecular structure is stabilized by an intramolecular C8—H8A···O1 hydrogen bond (Table 1) which generates an S(6) ring motif (Fig. 1; Bernstein *et al.*, 1995).

In the crystal structure (Fig. 2), the molecules are interconnected by C3—H3A···O2 hydrogen bonds (Table 1), forming a dimer with an *R*_2_^2^(8) ring motif. These dimers are further linked by intermolecular C17—H17A···O5 hydrogen bonds into an infinite chains along the [011] direction.

### Experimental

To a mixture of 2,5-dimethoxy acetophenone (1.5 ml, 0.01 mol) and 3-nitrobenzaldehyde (1.51 g, 0.01 mol) in ethanol (50 ml), 10 ml of 10% sodium hydroxide solution was added and stirred at 5–10 °C for 3 h. The precipitate formed was collected by filtration and then purified by recrystallization from ethanol. The single crystals were grown from a DMF solution by slow evaporation method (m.p. 377–379 K).

### Refinement

All H atoms were positioned geometrically (C—H = 0.93 or 0.96 Å) and refined using a riding model with *U*_iso_(H) = 1.2 or 1.5*U*_eq_(C). A rotating group model was applied to the methyl group.

### Crystal data

**Table:**

C_17_H_15_NO_5_	*Z* = 2
*M**_r_* = 313.30	*F*(000) = 328
Triclinic, *P*1	*D*_x_ = 1.367 Mg m^−^^3^
Hall symbol: -P 1	Mo *K*α radiation, λ = 0.71073 Å
*a* = 7.5015 (5) Å	Cell parameters from 5160 reflections
*b* = 7.9962 (5) Å	θ = 2.6–32.3°
*c* = 13.2468 (8) Å	µ = 0.10 mm^−^^1^
α = 86.507 (1)°	*T* = 296 K
β = 80.342 (1)°	Block, yellow
γ = 76.332 (1)°	0.41 × 0.38 × 0.13 mm
*V* = 760.96 (8) Å^3^	

### Data collection

**Table:**

Bruker APEX DUO CCD area-detector diffractometer	4381 independent reflections
Radiation source: fine-focus sealed tube	3195 reflections with *I* > 2σ(*I*)
graphite	*R*_int_ = 0.021
φ and ω scans	θ_max_ = 30.0°, θ_min_ = 1.6°
Absorption correction: multi-scan (*SADABS*; Bruker, 2009)	*h* = −10→10
*T*_min_ = 0.960, *T*_max_ = 0.987	*k* = −11→11
16631 measured reflections	*l* = −18→18

### Refinement

**Table:**

Refinement on *F*^2^	Primary atom site location: structure-invariant direct methods
Least-squares matrix: full	Secondary atom site location: difference Fourier map
*R*[*F*^2^ > 2σ(*F*^2^)] = 0.048	Hydrogen site location: inferred from neighbouring sites
*wR*(*F*^2^) = 0.179	H-atom parameters constrained
*S* = 1.02	*w* = 1/[σ^2^(*F*_o_^2^) + (0.1091*P*)^2^ + 0.0754*P*] where *P* = (*F*_o_^2^ + 2*F*_c_^2^)/3
4381 reflections	(Δ/σ)_max_ < 0.001
210 parameters	Δρ_max_ = 0.25 e Å^−^^3^
0 restraints	Δρ_min_ = −0.22 e Å^−^^3^

### Special details

**Table:**

Geometry. All e.s.d.'s (except the e.s.d. in the dihedral angle between two l.s. planes) are estimated using the full covariance matrix. The cell e.s.d.'s are taken into account individually in the estimation of e.s.d.'s in distances, angles and torsion angles; correlations between e.s.d.'s in cell parameters are only used when they are defined by crystal symmetry. An approximate (isotropic) treatment of cell e.s.d.'s is used for estimating e.s.d.'s involving l.s. planes.
Refinement. Refinement of *F*^2^ against ALL reflections. The weighted *R*-factor *wR* and goodness of fit *S* are based on *F*^2^, conventional *R*-factors *R* are based on *F*, with *F* set to zero for negative *F*^2^. The threshold expression of *F*^2^ > σ(*F*^2^) is used only for calculating *R*-factors(gt) *etc*. and is not relevant to the choice of reflections for refinement. *R*-factors based on *F*^2^ are statistically about twice as large as those based on *F*, and *R*-factors based on ALL data will be even larger.

### Fractional atomic coordinates and isotropic or equivalent isotropic displacement parameters (Å^2^)

**Table:**

	*x*	*y*	*z*	*U*_iso_*/*U*_eq_	
O1	0.15506 (16)	0.16718 (11)	0.39750 (8)	0.0591 (3)	
O2	0.11277 (18)	−0.21224 (13)	0.07147 (7)	0.0671 (3)	
O3	0.3565 (2)	−0.36287 (12)	0.39765 (9)	0.0802 (4)	
O4	0.2174 (3)	0.37169 (16)	0.74170 (13)	0.0989 (5)	
O5	0.3301 (3)	0.3286 (2)	0.88284 (13)	0.1121 (6)	
N1	0.2941 (2)	0.2780 (2)	0.80490 (12)	0.0729 (4)	
C1	0.14468 (17)	0.07338 (14)	0.31743 (9)	0.0409 (3)	
C2	0.07436 (19)	0.15014 (15)	0.23001 (10)	0.0487 (3)	
H2A	0.0312	0.2690	0.2265	0.058*	
C3	0.0682 (2)	0.05257 (17)	0.14937 (10)	0.0502 (3)	
H3A	0.0228	0.1057	0.0914	0.060*	
C4	0.12955 (19)	−0.12516 (16)	0.15424 (9)	0.0458 (3)	
C5	0.19828 (17)	−0.20273 (14)	0.23995 (9)	0.0419 (3)	
H5A	0.2391	−0.3219	0.2429	0.050*	
C6	0.20797 (16)	−0.10584 (13)	0.32289 (8)	0.0381 (2)	
C7	0.29087 (19)	−0.20926 (14)	0.40881 (9)	0.0452 (3)	
C8	0.2943 (2)	−0.12935 (16)	0.50575 (9)	0.0497 (3)	
H8A	0.2364	−0.0137	0.5148	0.060*	
C9	0.37566 (19)	−0.21468 (15)	0.57977 (9)	0.0460 (3)	
H9A	0.4334	−0.3300	0.5689	0.055*	
C10	0.38354 (17)	−0.14415 (15)	0.67833 (8)	0.0419 (3)	
C11	0.33505 (18)	0.03198 (16)	0.69527 (9)	0.0455 (3)	
H11A	0.2967	0.1094	0.6436	0.055*	
C12	0.34453 (19)	0.09026 (18)	0.78952 (10)	0.0522 (3)	
C13	0.3995 (2)	−0.0188 (2)	0.86864 (11)	0.0644 (4)	
H13A	0.4044	0.0240	0.9316	0.077*	
C14	0.4467 (2)	−0.1924 (2)	0.85189 (11)	0.0685 (4)	
H14A	0.4839	−0.2685	0.9044	0.082*	
C15	0.4399 (2)	−0.25623 (18)	0.75773 (10)	0.0542 (3)	
H15A	0.4732	−0.3743	0.7476	0.065*	
C16	0.0965 (3)	0.34867 (17)	0.39303 (15)	0.0703 (5)	
H16A	0.1164	0.3956	0.4541	0.105*	
H16B	−0.0333	0.3808	0.3876	0.105*	
H16C	0.1665	0.3928	0.3344	0.105*	
C17	0.1863 (3)	−0.3918 (2)	0.07001 (13)	0.0749 (5)	
H17A	0.1741	−0.4357	0.0063	0.112*	
H17B	0.1195	−0.4461	0.1256	0.112*	
H17C	0.3152	−0.4158	0.0772	0.112*	

### Atomic displacement parameters (Å^2^)

**Table:**

	*U*^11^	*U*^22^	*U*^33^	*U*^12^	*U*^13^	*U*^23^
O1	0.0856 (7)	0.0345 (4)	0.0589 (6)	−0.0060 (4)	−0.0224 (5)	−0.0149 (4)
O2	0.0998 (9)	0.0582 (6)	0.0452 (5)	−0.0064 (5)	−0.0298 (5)	−0.0119 (4)
O3	0.1363 (12)	0.0367 (5)	0.0692 (7)	0.0086 (6)	−0.0573 (7)	−0.0125 (4)
O4	0.1368 (14)	0.0526 (7)	0.1063 (11)	−0.0103 (8)	−0.0283 (10)	−0.0141 (7)
O5	0.1285 (13)	0.1081 (11)	0.1059 (11)	−0.0205 (10)	−0.0210 (10)	−0.0706 (9)
N1	0.0739 (9)	0.0682 (8)	0.0789 (9)	−0.0184 (7)	−0.0019 (7)	−0.0394 (7)
C1	0.0446 (6)	0.0331 (5)	0.0452 (6)	−0.0076 (4)	−0.0069 (4)	−0.0070 (4)
C2	0.0549 (7)	0.0347 (5)	0.0548 (7)	−0.0055 (5)	−0.0117 (5)	0.0014 (5)
C3	0.0566 (7)	0.0475 (6)	0.0456 (6)	−0.0065 (5)	−0.0153 (5)	0.0041 (5)
C4	0.0543 (7)	0.0467 (6)	0.0374 (5)	−0.0092 (5)	−0.0113 (5)	−0.0068 (4)
C5	0.0517 (7)	0.0342 (5)	0.0396 (5)	−0.0050 (4)	−0.0113 (5)	−0.0071 (4)
C6	0.0440 (6)	0.0332 (5)	0.0374 (5)	−0.0066 (4)	−0.0085 (4)	−0.0057 (4)
C7	0.0595 (7)	0.0365 (5)	0.0418 (6)	−0.0074 (5)	−0.0172 (5)	−0.0058 (4)
C8	0.0688 (8)	0.0402 (6)	0.0402 (6)	−0.0060 (5)	−0.0158 (5)	−0.0082 (4)
C9	0.0570 (7)	0.0386 (5)	0.0439 (6)	−0.0076 (5)	−0.0149 (5)	−0.0072 (4)
C10	0.0441 (6)	0.0451 (6)	0.0376 (5)	−0.0091 (5)	−0.0100 (4)	−0.0048 (4)
C11	0.0509 (7)	0.0473 (6)	0.0404 (6)	−0.0124 (5)	−0.0090 (5)	−0.0070 (4)
C12	0.0499 (7)	0.0577 (7)	0.0500 (7)	−0.0112 (6)	−0.0058 (5)	−0.0204 (6)
C13	0.0604 (9)	0.0892 (11)	0.0426 (7)	−0.0066 (8)	−0.0139 (6)	−0.0193 (7)
C14	0.0716 (10)	0.0849 (11)	0.0427 (7)	0.0005 (8)	−0.0199 (6)	0.0043 (7)
C15	0.0583 (8)	0.0526 (7)	0.0484 (7)	−0.0022 (6)	−0.0151 (6)	0.0014 (5)
C16	0.0901 (12)	0.0352 (6)	0.0849 (11)	−0.0072 (7)	−0.0160 (9)	−0.0185 (7)
C17	0.1071 (14)	0.0604 (9)	0.0567 (8)	−0.0066 (9)	−0.0215 (8)	−0.0245 (7)

### Geometric parameters (Å, °)

**Table:**

O1—C1	1.3585 (13)	C8—H8A	0.9300
O1—C16	1.4140 (15)	C9—C10	1.4689 (15)
O2—C4	1.3725 (13)	C9—H9A	0.9300
O2—C17	1.4114 (18)	C10—C11	1.3911 (16)
O3—C7	1.2186 (14)	C10—C15	1.3941 (16)
O4—N1	1.214 (2)	C11—C12	1.3775 (16)
O5—N1	1.2241 (18)	C11—H11A	0.9300
N1—C12	1.477 (2)	C12—C13	1.375 (2)
C1—C2	1.4006 (17)	C13—C14	1.372 (2)
C1—C6	1.4007 (14)	C13—H13A	0.9300
C2—C3	1.3736 (18)	C14—C15	1.3894 (19)
C2—H2A	0.9300	C14—H14A	0.9300
C3—C4	1.3876 (17)	C15—H15A	0.9300
C3—H3A	0.9300	C16—H16A	0.9600
C4—C5	1.3781 (16)	C16—H16B	0.9600
C5—C6	1.4035 (14)	C16—H16C	0.9600
C5—H5A	0.9300	C17—H17A	0.9600
C6—C7	1.4986 (15)	C17—H17B	0.9600
C7—C8	1.4754 (15)	C17—H17C	0.9600
C8—C9	1.3121 (17)		
			
C1—O1—C16	119.72 (11)	C10—C9—H9A	117.1
C4—O2—C17	117.66 (10)	C11—C10—C15	118.74 (11)
O4—N1—O5	124.18 (17)	C11—C10—C9	121.88 (10)
O4—N1—C12	118.90 (13)	C15—C10—C9	119.38 (11)
O5—N1—C12	116.90 (18)	C12—C11—C10	119.18 (12)
O1—C1—C2	122.18 (10)	C12—C11—H11A	120.4
O1—C1—C6	118.48 (10)	C10—C11—H11A	120.4
C2—C1—C6	119.33 (10)	C13—C12—C11	122.74 (13)
C3—C2—C1	121.03 (11)	C13—C12—N1	119.30 (13)
C3—C2—H2A	119.5	C11—C12—N1	117.96 (13)
C1—C2—H2A	119.5	C14—C13—C12	118.00 (12)
C2—C3—C4	120.09 (11)	C14—C13—H13A	121.0
C2—C3—H3A	120.0	C12—C13—H13A	121.0
C4—C3—H3A	120.0	C13—C14—C15	121.00 (13)
O2—C4—C5	124.45 (11)	C13—C14—H14A	119.5
O2—C4—C3	115.96 (10)	C15—C14—H14A	119.5
C5—C4—C3	119.58 (10)	C14—C15—C10	120.34 (13)
C4—C5—C6	121.45 (10)	C14—C15—H15A	119.8
C4—C5—H5A	119.3	C10—C15—H15A	119.8
C6—C5—H5A	119.3	O1—C16—H16A	109.5
C1—C6—C5	118.51 (10)	O1—C16—H16B	109.5
C1—C6—C7	126.70 (9)	H16A—C16—H16B	109.5
C5—C6—C7	114.78 (9)	O1—C16—H16C	109.5
O3—C7—C8	119.73 (10)	H16A—C16—H16C	109.5
O3—C7—C6	118.73 (10)	H16B—C16—H16C	109.5
C8—C7—C6	121.54 (10)	O2—C17—H17A	109.5
C9—C8—C7	122.75 (11)	O2—C17—H17B	109.5
C9—C8—H8A	118.6	H17A—C17—H17B	109.5
C7—C8—H8A	118.6	O2—C17—H17C	109.5
C8—C9—C10	125.75 (11)	H17A—C17—H17C	109.5
C8—C9—H9A	117.1	H17B—C17—H17C	109.5
			
C16—O1—C1—C2	−1.1 (2)	C5—C6—C7—C8	−173.92 (12)
C16—O1—C1—C6	178.45 (13)	O3—C7—C8—C9	4.2 (2)
O1—C1—C2—C3	178.92 (12)	C6—C7—C8—C9	−175.88 (13)
C6—C1—C2—C3	−0.6 (2)	C7—C8—C9—C10	−179.56 (12)
C1—C2—C3—C4	1.0 (2)	C8—C9—C10—C11	−12.8 (2)
C17—O2—C4—C5	−6.0 (2)	C8—C9—C10—C15	166.73 (14)
C17—O2—C4—C3	175.36 (15)	C15—C10—C11—C12	0.15 (19)
C2—C3—C4—O2	178.06 (13)	C9—C10—C11—C12	179.72 (12)
C2—C3—C4—C5	−0.6 (2)	C10—C11—C12—C13	−0.3 (2)
O2—C4—C5—C6	−178.65 (12)	C10—C11—C12—N1	179.37 (12)
C3—C4—C5—C6	−0.1 (2)	O4—N1—C12—C13	−168.10 (17)
O1—C1—C6—C5	−179.64 (11)	O5—N1—C12—C13	10.5 (2)
C2—C1—C6—C5	−0.08 (18)	O4—N1—C12—C11	12.2 (2)
O1—C1—C6—C7	−0.96 (19)	O5—N1—C12—C11	−169.16 (15)
C2—C1—C6—C7	178.60 (12)	C11—C12—C13—C14	0.1 (2)
C4—C5—C6—C1	0.41 (19)	N1—C12—C13—C14	−179.54 (15)
C4—C5—C6—C7	−178.42 (12)	C12—C13—C14—C15	0.2 (3)
C1—C6—C7—O3	−172.69 (14)	C13—C14—C15—C10	−0.4 (2)
C5—C6—C7—O3	6.03 (19)	C11—C10—C15—C14	0.2 (2)
C1—C6—C7—C8	7.4 (2)	C9—C10—C15—C14	−179.40 (13)

### Hydrogen-bond geometry (Å, °)

**Table:**

*D*—H···*A*	*D*—H	H···*A*	*D*···*A*	*D*—H···*A*
C3—H3A···O2^i^	0.93	2.55	3.4773 (18)	172.
C8—H8A···O1	0.93	2.12	2.7727 (16)	126.
C17—H17A···O5^ii^	0.96	2.50	3.309 (2)	142.

Symmetry codes: (i) −*x*, −*y*, −*z*; (ii) *x*, *y*−1, *z*−1.
